# Trends in the prevalence of anorexia nervosa among individuals under 45 years in China, 1992–2021: an age-period-cohort analysis

**DOI:** 10.3389/fpubh.2025.1634147

**Published:** 2025-08-25

**Authors:** Jun Tian, Yuheng He, Yiyi Chen, Zheng Tang, Yongzhao Zhou

**Affiliations:** ^1^Mental Health Center, West China Hospital, Sichuan University, Chengdu, China; ^2^Integrated Care Management Center, Outpatient Department, West China Hospital, Sichuan University, Chengdu, China

**Keywords:** anorexia nervosa, epidemiological trends, age-period-cohort model, GBD, GBD database

## Abstract

**Objective:**

This study aims to analyze the temporal trends in the incidence and prevalence of anorexia nervosa (AN) in China from 1992 to 2021, focusing on age, period, and cohort effects, in order to provide evidence for the prevention and control of anxiety-related disorders.

**Methods:**

Data on the incidence and prevalence of anorexia nervosa in China were obtained from the Global Burden of Disease Study 2021. Joinpoint regression analysis was employed to assess trends over time, while an age-period-cohort (APC) model was used to estimate the net effects of age, period, and cohort variables.

**Results:**

From 1992 to 2021, the incidence and prevalence of anorexia nervosa among individuals under 45 years in China showed a general upward trend. The annual percent change (APC) for incidence was 1.29%, and for prevalence was 0.31%, with both rates consistently higher in females than in males. Age effects revealed that the highest incidence and prevalence occurred between ages 10 and 25, followed by a gradual decline. Period effects demonstrated a consistent upward trend across the total population. Cohort analysis indicated an intergenerational increase in the risk of anorexia nervosa.

**Conclusion:**

The incidence and prevalence of anorexia nervosa among individuals under 45 years in China have increased steadily from 1992 to 2021. This growing disease burden, with marked age and gender disparities, highlights the urgent need for targeted interventions, especially among adolescents and women.

## Background

Anorexia nervosa (AN) is a severe psychiatric disorder characterized by extreme food restriction and pathological control of body weight, classified under eating disorders. Patients often exhibit an intense fear of weight gain, resulting in chronic malnutrition. Classic symptoms include a significantly low body mass index (BMI < 17.5), menstrual irregularities in females, and denial of the harmful consequences of being underweight (1, 2). AN is frequently accompanied by multisystem complications, such as cardiac abnormalities (3) and increased bone fragility (2), and it is highly comorbid with other mental disorders, including depression, anxiety, and obsessive-compulsive disorder (4). Although approximately half of patients can achieve partial or full recovery with treatment, up to 5% die from organ failure or suicide, and 20% develop a chronic disease course, posing serious threats to health (2).

This disorder is especially prevalent among young individuals. Over the past three decades, the global prevalence of AN has increased significantly. According to global epidemiological data from 2017, the age-standardized prevalence rates were 7.51 per 100,000 in males and 22.46 per 100,000 in females, with adolescent girls aged 15–19 being most affected (5, 6). In China, the number of affected adolescents has also risen, with urbanized areas such as Hong Kong approaching prevalence rates comparable to those in Western developed countries (7). In addition to severely impairing quality of life, AN markedly increases the risks of substance abuse and suicide, and it has the highest mortality rate among all psychiatric conditions (8), placing a substantial burden on healthcare systems (9).

However, there remains a lack of comprehensive studies on the temporal trends of AN among individuals under the age of 45 in China. Most existing studies have only described age-specific incidence or prevalence at discrete time points, without adequately disentangling the interacting effects of age, period, and birth cohort (APC). This limitation hinders the identification of high-risk populations and key exposures. To address this gap, the present study uses data from the Global Burden of Disease Study 2021 (GBD 2021) to systematically analyze trends in the prevalence of AN in China from 1992 to 2021. By quantifying the independent effects of age, period, and cohort, this study aims to provide scientific evidence for the development of prevention and intervention strategies throughout the life course.

## Methods

### Data source

The data for this study were obtained from the Global Burden of Disease Study 2021 (GBD 2021) database, maintained by the Institute for Health Metrics and Evaluation (IHME). This database provides comprehensive observational epidemiological data for 369 diseases and injuries across 204 countries and territories from 1992 to 2021 (10). The AN data for China were derived from multiple sources, including disease surveillance systems, national health service surveys, and published literature. This study extracted data on the incidence and prevalence of AN among individuals aged under 45 in China from 1992 to 2021 for descriptive and analytical purposes.

### Joinpoint regression analysis

Joinpoint regression analysis involves segmented linear modeling and permutation tests to objectively identify turning points in time-series data, such as critical public health intervention points. It overcomes the limitations of traditional linear regression in fitting complex non-linear patterns (e.g., fluctuating incidence rates or economic cycles). The method’s strengths include automated model selection (11), statistically validated identification of joinpoints (with Bayesian Information Criterion for overfitting control), and the ability to adjust for covariates, enabling multidimensional stratified analysis (12). Developed by the National Cancer Institute (NCI), this technique has been widely applied in cancer epidemiology (e.g., trend decomposition in survival rates) and environmental exposure assessment (e.g., climate change inflection points). Its visual outputs offer intuitive evidence to inform policy decisions (13).

### Age-period-cohort analysis

Age-period-cohort (APC) analysis is a statistical approach designed to separate the independent effects of age (biological/life stage), period (contemporary historical events), and birth cohort (shared generational experiences) on a given outcome (14). This method is particularly effective in disentangling complex temporal dynamics. The age effect reflects biological or lifecycle-driven variations in risk (e.g., age-related disease vulnerability), period effects capture time-specific global influences (e.g., health policies or pandemics), and cohort effects reveal generational differences rooted in distinct social environments (e.g., shifts in health behaviors or cultural attitudes). Linear collinearity among the three dimensions is addressed via constrained estimators (e.g., the Intrinsic Estimator) or hierarchical modeling techniques (15). APC analysis is widely used in public health for identifying high-risk groups and guiding targeted interventions (e.g., cancer screening by birth cohort) and in sociology to study generational value shifts.

### Statistical analysis

Key analytical indicators in this study included age-standardized incidence rates (ASIR) and age-standardized prevalence rates (ASPR) of anorexia nervosa. The study population included individuals aged 5–45 years from 1992 to 2021 in China. The population was stratified into eight 5-year age groups (5–9, 10–14, 15–19, 20–24, 25–29, 30–34, 35–39, 40–44), six period groups (1992–1996, 1997–2001, 2002–2006, 2007–2011, 2012–2016, 2017–2021), and 13 birth cohorts (1948–1952 to 2013–2017). The 2002–2006 period and the 1982–1986 birth cohort were designated as reference categories. Joinpoint regression was performed using the JPR software (version 5.0.2) developed by the U. S. National Cancer Institute. APC analyses were conducted using the NCI’s online APC analysis tool (11).

## Results

**Figure 1:**
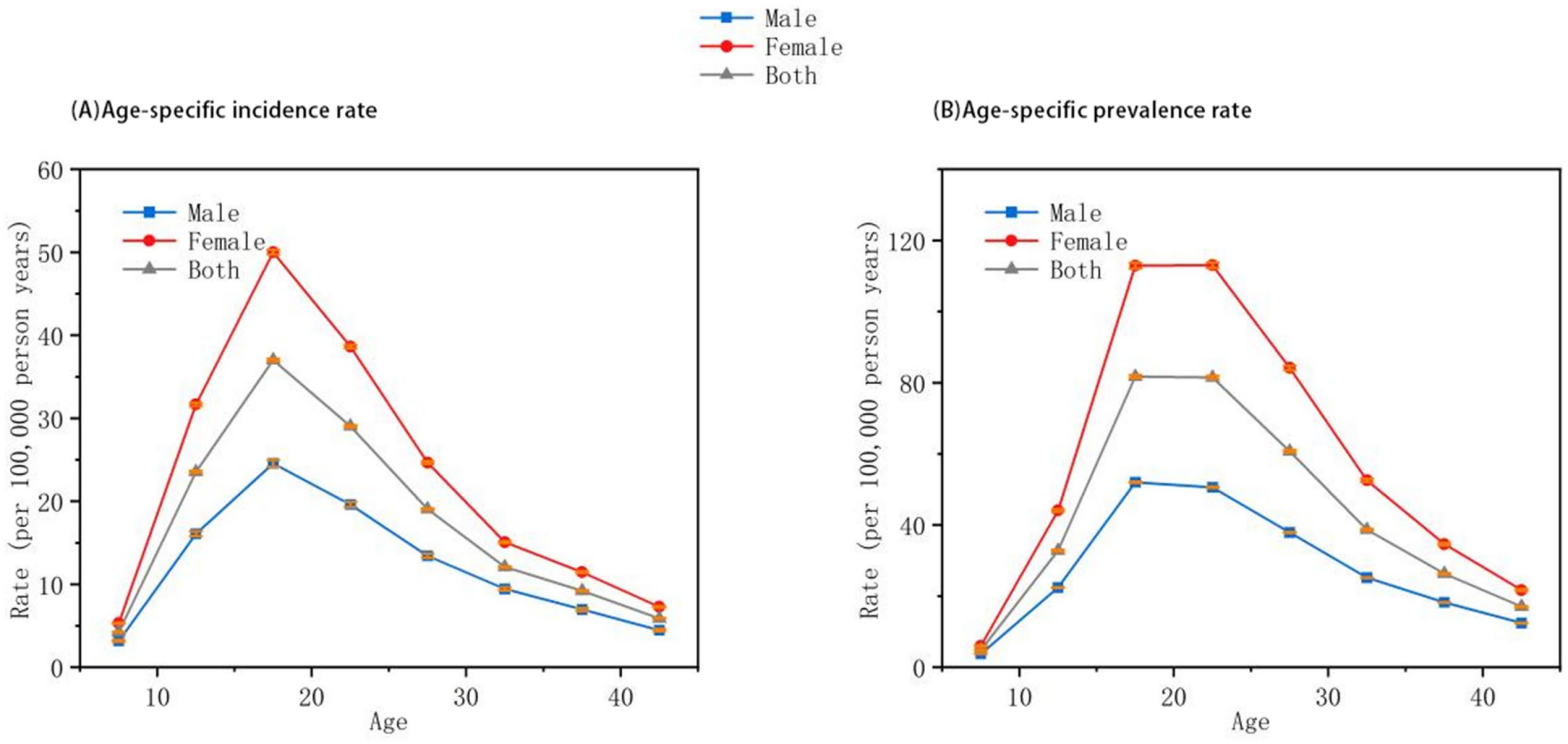
Joinpoint regression analysis of anorexia nervosa incidence and prevalence in China among individuals aged under 45 years (1992–2021). **(A)** Age-standardized incidence rates. **(B)** Age-standardized prevalence rates. Data Source: Global Burden of Disease Study 2021 (GBD 2021). Trends are segmented based on statistically significant inflection points identified through Joinpoint regression. All trends shown are statistically significant (*p* < 0.05).

Trends in Incidence and Prevalence of Anorexia Nervosa Among Individuals Under 45 Years in China (1992–2021). Table 1 and Figure 1A present the Joinpoint regression analysis of the age-standardized incidence rates (ASIR) of anorexia nervosa among individuals under 45 years in China from 1992 to 2021. The overall trend, as well as the trends in males and females, demonstrated significant segmented increases. On average, the incidence rate increased by 1.29% per year, with annual increases of 1.25% in males and 1.37% in females. Each group experienced six distinct segments of upward trends, all of which were statistically significant (*p* < 0.05). The steepest increase in the overall population occurred between 2009 and 2014, with an annual rise of 2.32%. Among males, the fastest increase was observed from 2006 to 2014 (1.69% per year), while in females, the most pronounced growth occurred from 2010 to 2015 (2.78% per year). The differences in trend slopes between males and females were statistically significant (*p* < 0.05).

**Table 1:** Joinpoint regression analysis of anorexia nervosa incidence rates by sex in China among individuals aged under 45 years (1992–2021).

Category	Indicator	Interval	Change (%)	95% CI
Overall	APC	1992–1996	0.5270*	0.2504 ~ 0.6818
		1996–1999	1.8384*	1.5831 ~ 1.9899
		1999–2009	1.2490*	1.1778 ~ 1.2882
		2009–2014	2.3251*	2.1921 ~ 2.5438
		2014–2017	1.3072*	1.0195 ~ 1.8156
		2017–2021	0.4706*	0.2264 ~ 0.6186
	AAPC	1992–2021	1.2927*	1.2721 ~ 1.3106
Male	APC	1992–1996	0.5283*	0.4866 ~ 0.5720
		1996–2002	1.2172*	1.1704 ~ 1.2456
		2002–2006	1.4033*	1.3186 ~ 1.5167
		2006–2014	1.6857*	1.6660 ~ 1.7268
		2014–2018	1.5262*	1.4614 ~ 1.5762
		2018–2021	0.5837*	0.5198 ~ 0.6442
	AAPC	1992–2021	1.2533*	1.2474 ~ 1.2591
Female		1992–1996	0.5527*	0.2329 ~ 0.7414
		1996–2000	2.0285*	1.8097 ~ 2.3375
		2000–2006	1.0232*	0.7534 ~ 1.1531
		2006–2010	1.5991*	1.2745 ~ 2.2778
		2010–2015	2.7846*	2.6408 ~ 3.0261
		2015–2021	0.5308*	0.4123 ~ 0.6307
	AAPC	1992–2021	1.3749*	1.3535 ~ 1.3959

Annual percent change (APC) and average annual percent change (AAPC) are presented for each identified segment. *All APCs are statistically significant at *p* < 0.05.

Table 2 and Figure 1B show the Joinpoint regression results for age-standardized prevalence rates (ASPR). From 1992 to 2021, the prevalence of anorexia nervosa also showed significant segmented increases in the overall population, as well as among males and females. The average annual increase in ASPR was 1.34% overall, 1.31% in males, and 1.40% in females. Both the overall population and females exhibited six periods of increasing prevalence, whereas four distinct increasing periods were identified in males, all with statistical significance (*p* < 0.05). The most substantial increases in the overall and female populations occurred between 1996 and 2000, with annual growth rates of 2.18 and 2.53%, respectively. For males, the largest increase occurred between 2004 and 2018 (1.68% annually). The differences in prevalence trends between sexes were also statistically significant (*p* < 0.05).

**Table 2:** Joinpoint regression analysis of anorexia nervosa prevalence rates by sex in China among individuals aged under 45 years (1992–2021).

Category	Indicator	Interval	Change (%)	95% CI
Overall	APC	1992–1996	0.5651*	0.4876 ~ 0.6432
		1996–2000	2.1761*	2.1057 ~ 2.2491
		2000–2005	1.2096*	1.1150 ~ 1.2811
		2005–2013	1.7129*	1.6719 ~ 1.7751
		2013–2018	1.3550*	1.2542 ~ 1.4347
		2018–2021	0.4548*	0.3319 ~ 0.5830
	AAPC	1992–2021	1.3384*	1.3271 ~ 1.3491
Male	APC	1992–1996	0.4590*	0.3897 ~ 0.5347
		1996–2004	1.3574*	1.3084 ~ 1.3923
		2004–2018	1.6810*	1.6653 ~ 1.7009
		2018–2021	0.6262*	0.5212 ~ 0.7126
	AAPC	1992–2021	1.3130*	1.3024 ~ 1.3233
Female	APC	1992–1996	0.5957*	0.4974 ~ 0.6957
		1996–2000	2.5337*	2.4462 ~ 2.6259
		2000 ~ 2005	1.0581*	0.9534 ~ 1.1451
		2005 ~ 2014	1.7735*	1.7319 ~ 1.8374
		2014 ~ 2018	1.3751*	1.1719 ~ 1.5303
		2018 ~ 2021	0.5094*	0.2940 ~ 0.7001
	AAPC	1992 ~ 2021	1.4048*	1.3902 ~ 1.4182

Annual percent change (APC) and average annual percent change (AAPC) are presented for each identified segment. *All APCs are statistically significant at *P* < 0.05.

### Temporal effects analysis: age, period, and cohort dimensions

**Figure 2:**
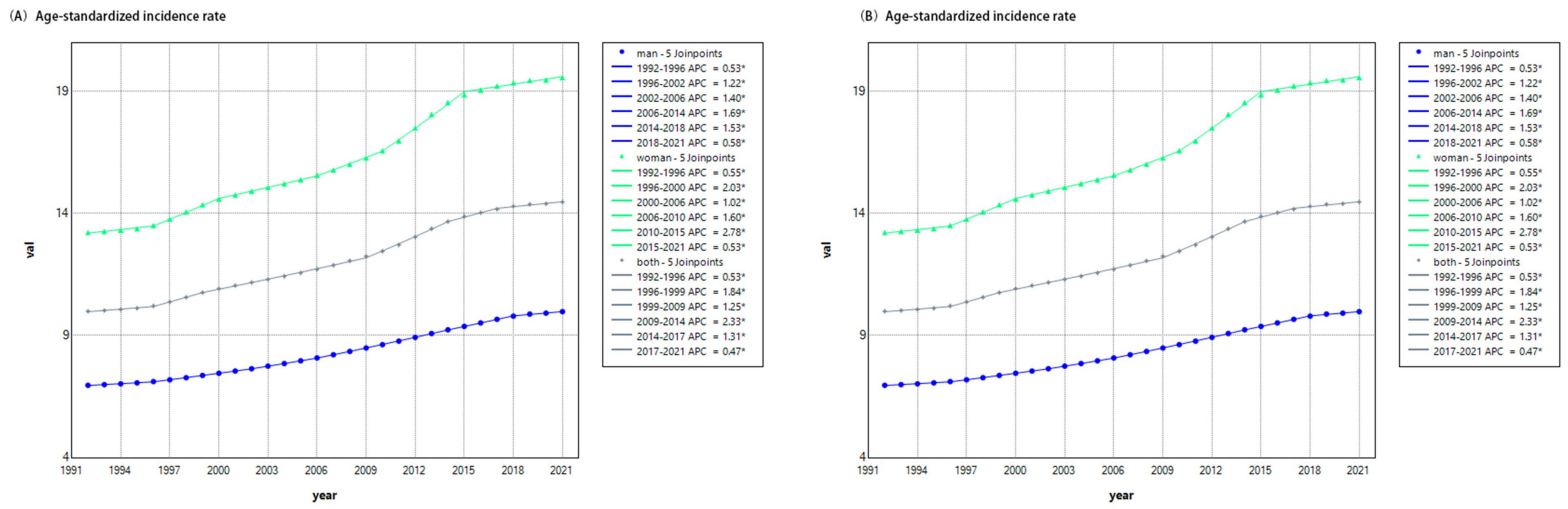
Longitudinal age effects on anorexia nervosa among individuals aged under 45 years in China (1992–2021). **(A)** Age-specific incidence rates. **(B)** Age-specific prevalence rates. Data adjusted for period effects. Both incidence and prevalence rates peak between ages 10 and 25 and decline thereafter. Females exhibit higher rates than males across all age groups.

The age-period-cohort (APC) analysis revealed distinct and interrelated contributions of age, period, and cohort effects to the observed trends in anorexia nervosa (AN) among individuals under 45 years in China from 1992 to 2021. The age effect demonstrated a pronounced pattern (Figure 2): both incidence and prevalence peaked between the ages of 10 and 25, followed by a gradual decline. This trend was consistent in both sexes, though females exhibited higher rates across all age groups. These findings underscore adolescence and early adulthood as high-risk developmental windows, characterized by hormonal fluctuations, emerging self-identity, and heightened sensitivity to social influences—all of which may predispose individuals to disordered eating behaviors.

**Figure 3:**
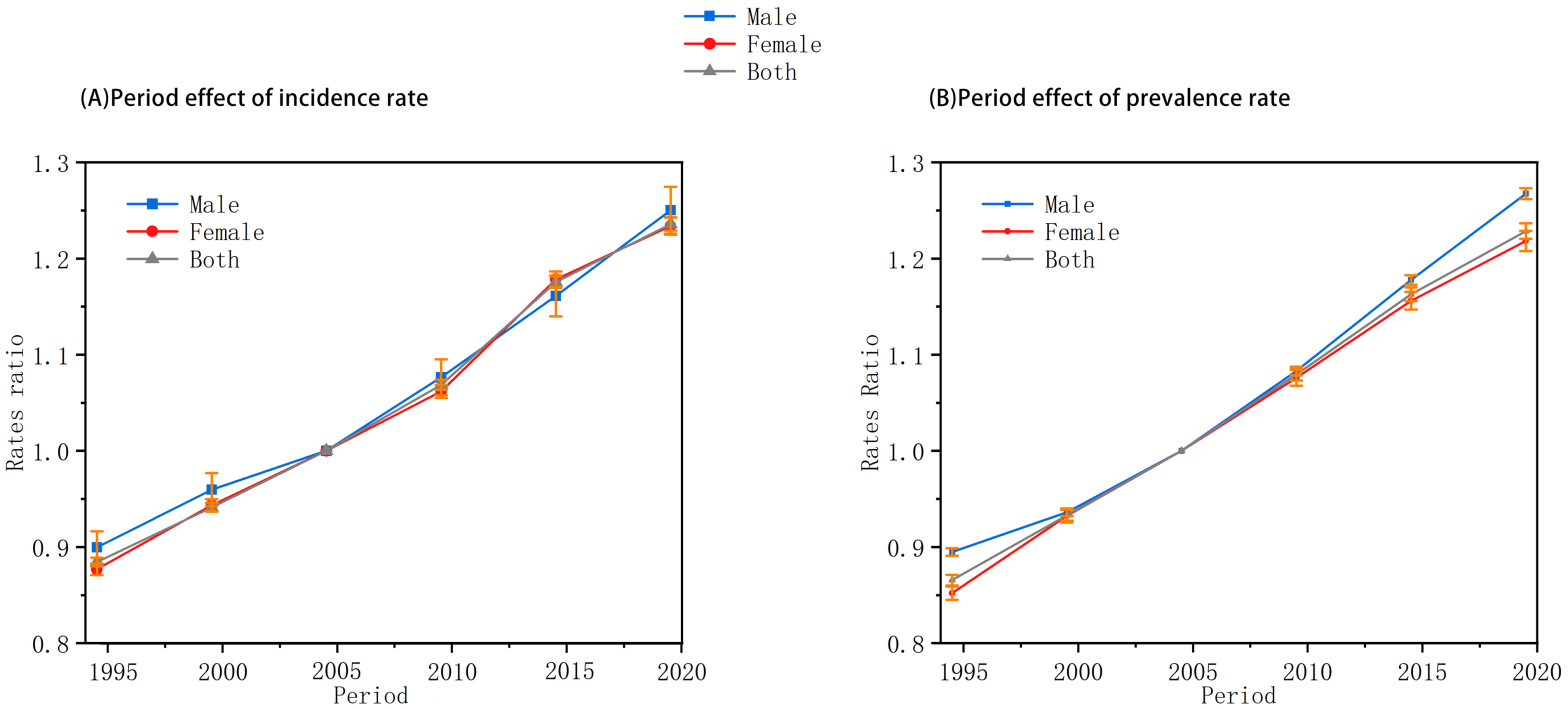
Period effects on anorexia nervosa incidence and prevalence in China among individuals aged under 45 years (1992–2021). **(A)** Relative risks of incidence by period. **(B)** Relative risks of prevalence by period. Relative risks (RRs) adjusted for age and cohort effects. Period effects demonstrate a steady increase over time. Gender differences are observed, particularly in the later periods (2015–2021).

The period effect (Figure 3) showed a persistent upward trend in the relative risk (RR) of both incidence and prevalence over time, independent of age and cohort influences. Between 1992 and 2010, males exhibited higher incidence risks compared to females; however, by 2015–2021, the prevalence risk in males surpassed that in females. This pattern reflects broad sociocultural and technological shifts during the study period—including the proliferation of media promoting thin ideals, increased social media use, and intensified academic and workplace pressures—that likely contributed to body dissatisfaction and restrictive eating across the population.

**Figure 4:**
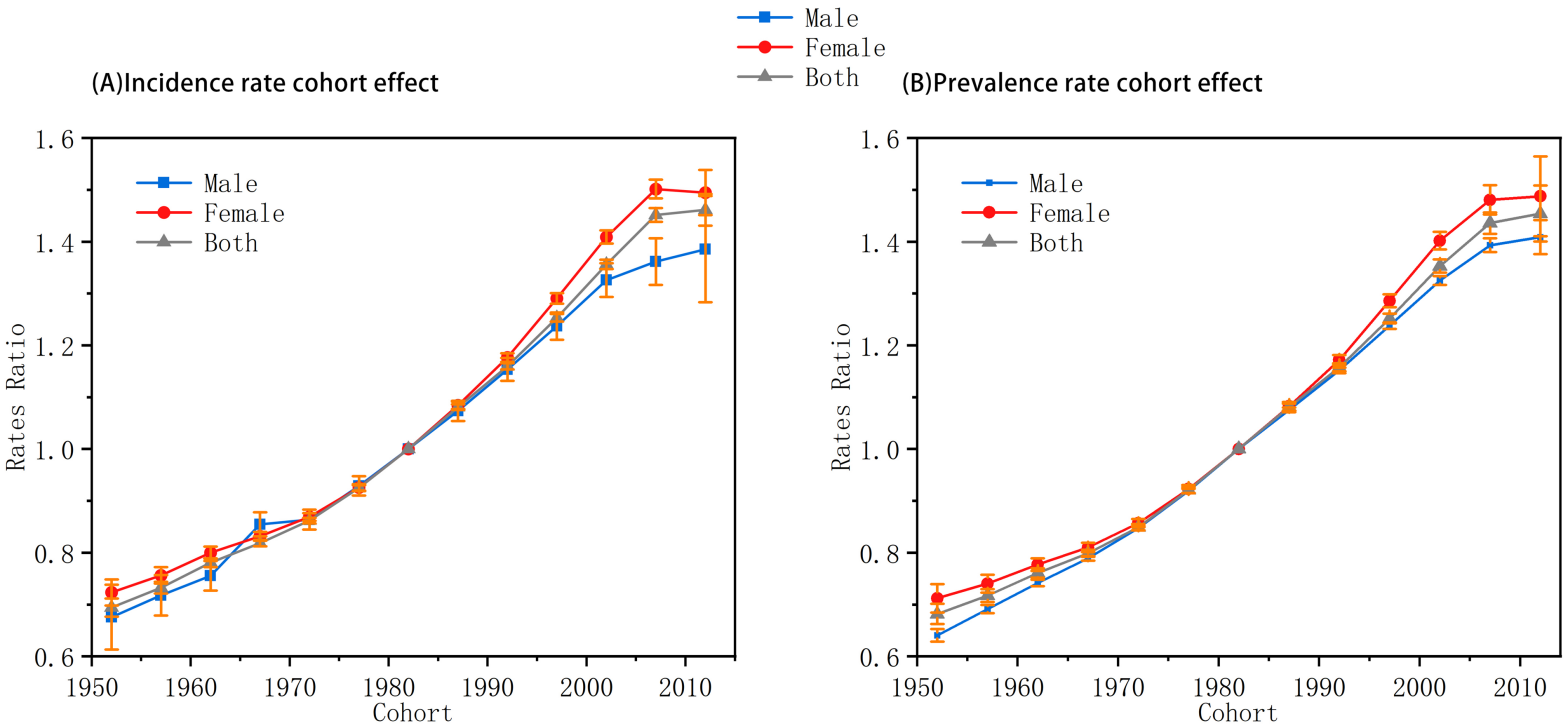
Cohort effects on anorexia nervosa incidence and prevalence in China among individuals aged under 45 years (1992–2021). **(A)** Relative risks of incidence by birth cohort. **(B)** Relative risks of prevalence by birth cohort. Relative risks (RRs) adjusted for age and period effects. A consistent upward trend in RR is observed among more recent birth cohorts. No significant gender difference is detected in cohort trends.

The cohort effect (Figure 4) illustrated a generational rise in AN risk, with RR values steadily increasing among later-born cohorts. This trend suggests that individuals born more recently are more vulnerable to AN, likely due to cumulative exposure to modern stressors such as digital media, urbanization, and societal competition. Notably, the cohort effect showed no significant gender differences, indicating that these generational pressures may exert similarly adverse influences on both males and females. Together, these temporal dimensions reflect a complex interplay of biological, cultural, and environmental forces driving the increasing burden of AN in younger populations.

## Discussion

To our knowledge, this study is the first to comprehensively assess the trends in incidence and prevalence of anorexia nervosa (AN) among individuals under 45 years in China using GBD 2021 data from 1992 to 2021. Over the past three decades, both the incidence and prevalence of AN in this population have steadily increased. Specifically, the incidence rate rose from 10 per 100,000 in 1992 to 14.48 per 100,000 in 2021, while the prevalence increased from 22.98 to 32.81 per 100,000. The age-period-cohort (APC) model revealed that all three temporal dimensions contributed significantly to these trends, with age emerging as a particularly influential factor.

The age effect showed a distinct gender difference, with females consistently exhibiting higher incidence and prevalence rates across all age groups. The highest burden of AN was concentrated in individuals aged 10–25, after which both incidence and prevalence declined gradually. These findings align with the results of previous studies by Li et al. (16) and Wu et al. (6). In addition, this study limited the upper age boundary to 45 years because AN predominantly affects adolescents and young adults. Epidemiological studies have demonstrated that the prevalence and incidence of AN markedly decline after age 45 (17). Moreover, the GBD 2021 dataset primarily emphasizes younger populations, especially ages 10–25, where AN risk is highest, thus focusing on this age range allows for a clearer depiction of trends in the most affected groups. It should be noted that the DSM-5 (2013) broadened the diagnostic definition of AN by removing the amenorrhea criterion, thus including males who may previously have been excluded. As GBD data span 1992–2021, crossing the DSM-IV and DSM-5 eras, this diagnostic shift likely contributed to the observed increase in incidence and prevalence, particularly after 2013, and should be considered when interpreting these findings (18). Adolescence is a period of profound physiological and psychological change. On a biological level, hormonal fluctuations—particularly in estrogen—may disrupt normal appetite regulation and affect body image perception in females (19). Psychologically, adolescents are in the midst of forming self-identity and are highly sensitive to external influences (20). Socially, the increasing importance of peer acceptance and exposure to societal norms around body image can lead to unhealthy dieting behaviors and ultimately the development of eating disorders (21).

Our results also confirm a marked gender disparity: females exhibit significantly higher rates of AN than males, consistent with international trends (4). Compared with international studies, our reported AN incidence in China is higher. This discrepancy may stem from several factors: GBD data integrate multiple sources including surveillance, surveys, and literature, which may encompass broader definitions or self-reported cases. Additionally, China’s rapid socioeconomic changes and cultural westernization could have elevated true incidence, and differences in diagnostic rigor between studies also likely contributed (22, 23). Socioculturally, the widespread promotion of thinness as a beauty ideal in China—reinforced by fashion media and social platforms—places substantial body image pressure on women. In pursuit of this “ideal body,” women are more likely to engage in extreme dietary restrictions, thereby elevating their risk of developing AN (24, 25). Psychologically, women tend to construct self-identity more strongly around physical appearance. Dissatisfaction with one’s body or weight may lead to emotional distress and, in turn, trigger restrictive eating as a coping mechanism to alleviate anxiety and reinforce self-worth (17, 26).

China’s competitive education system imposes distinct institutional pressures on youth mental health, particularly during the critical developmental period between ages 10 and 20. Adolescents are often subjected to academic workloads exceeding 12 h per day, significantly reducing sleep compared to international standards. Coupled with high parental expectations—where education is often seen as the family’s primary investment—this dual burden can contribute to the onset of psychiatric disorders, including anorexia nervosa (27).

The period effect analysis revealed a steady increase in both incidence and prevalence ratios of AN from 1992 to 2021 across genders and in the total population. These findings reflect profound changes in China’s sociocultural and media landscape during this time.

From a cultural standpoint, the proliferation of the “thin ideal” through television, fashion media, and later social media platforms has amplified body image anxiety in both genders. He et al. (28) reported that extensive exposure to these ideals significantly elevates the risk of AN. As the internet became increasingly accessible during the study period, platforms such as Facebook and Weibo accelerated the viral spread of body-conscious norms. Notably, spending more than 3 h daily on short video platforms was associated with a 37% increase in the risk of AN.

Economically, the transition toward consumer-driven lifestyles has also contributed to this phenomenon. The rapid industrialization of fast food and the commercialization of the weight-loss industry since the 1990s created an environment of dietary paradox. Stice and Shaw (29) proposed the “affluence–restriction paradox,” whereby excessive availability of high-calorie foods coexists with pressure to maintain thinness. This paradox fosters increased dieting behavior among adolescents, a known precursor to AN. The co-occurrence of rising obesity and AN prevalence during the same period underscores a dual-pathway model of “weight anxiety → restrictive behavior.”

Cohort analysis further suggests that sociodemographic transitions have significantly influenced AN trends. The implementation of China’s one-child policy led to a dramatic increase in nuclear family structures, wherein emotional interactions between parents and children are often diminished. Reduced familial emotional support may prompt adolescents to seek control and comfort through eating behavior regulation. Restrictive eating can become a compensatory strategy to regain psychological control. These findings are consistent with global studies linking impaired family communication patterns to elevated risk of eating disorders (30, 31).

Moreover, China’s rapid urbanization has reshaped daily life and stress exposure across generations. Younger cohorts are increasingly immersed in fast-paced urban environments characterized by intense competition, workplace stress, and ongoing economic pressure. Chronic social stress, well-documented as a risk factor for psychiatric disorders—including AN—can disrupt normal eating patterns, leading individuals to adopt disordered eating as an ineffective coping strategy.

While this study emphasizes sociocultural influences, genetic factors are equally critical in AN etiology. Twin studies estimate heritability between 48 and 74%, and female relatives of AN patients have an 11-fold higher lifetime risk than those without such family history (32, 33). Future studies should integrate genetic and environmental perspectives to better elucidate AN’s multifactorial origins. Finally, the evolving economic climate has exacerbated employment stress among younger cohorts. The high competitiveness of the labor market often generates anxiety, depression, and other negative emotional states. In the absence of effective emotional regulation strategies, these emotions can manifest as maladaptive behaviors, including extreme dietary restriction. This pattern is consistent with previous findings identifying psychological stress and poor coping skills as major risk factors for the development of AN (34, 35).

Despite these findings, the study has several limitations. First, the GBD database provides only secondhand, aggregated data and currently includes data only up to 2021, which limits timeliness. Second, due to data availability constraints, regional analyses at the provincial or city level could not be performed. Third, the lack of individual-level disease burden data and standardized population information may lead to discrepancies between the reported estimates and the actual national situation in China. Given China’s pronounced socioeconomic and urban–rural disparities, future research should stratify by these dimensions. Urban residents’ greater exposure to social media and distinct body image norms versus rural populations could serve as an internal comparison to disentangle cultural from genetic effects. Future research should incorporate more granular, up-to-date data for enhanced accuracy and relevance.

## Conclusion

From 1992 to 2021, the incidence and prevalence of anorexia nervosa among individuals under 45 years in China have exhibited a persistent upward trend. The disease burden was especially high among females aged 10–25 years. Age-period-cohort analysis demonstrated that age was a major determinant, with incidence and prevalence peaking during adolescence and declining gradually thereafter. Meanwhile, differences between males and females narrowed with increasing age. Period effects showed a steady increase across the entire population, reflecting widespread societal changes. Cohort effects revealed a progressive intergenerational increase in AN risk, with no significant gender difference. These findings highlight the urgent need for sustained attention to AN among adolescents and women, alongside timely, targeted interventions to mitigate its growing public health impact.

## Data Availability

The original contributions presented in the study are included in the article/supplementary material, further inquiries can be directed to the corresponding author.
